# Optimization and Validation of an Ultra-Performance Liquid Chromatography with Quadrupole Detector Mass Spectrometry Quantification Method for the Simultaneous Detection of Tazarotene and Tazarotenic Acid in Porcine Skin: An In Vitro Study

**DOI:** 10.3390/ijms26020489

**Published:** 2025-01-09

**Authors:** Helena Hamzehpour, Kristófer H. Hauksson, Helgi Jónsson, Sveinbjorn Gizurarson, Bergthora S. Snorradottir

**Affiliations:** 1 Faculty of Pharmaceutical Sciences, University of Iceland, 107 Reykjavik, Iceland; heh83@hi.is (H.H.); sveinbj@hi.is (S.G.); 2 Faculty of Medicine, University of Iceland, 107 Reykjavik, Iceland; helgi@hi.is

**Keywords:** tazarotene, tazarotenic acid, hand osteoarthritis, quadrupole detector mass spectrometry (QDa), design of experiments (DoE), transdermal, bioanalytical techniques

## Abstract

Exploring tazarotene, a third-generation retinoid for potential hand osteoarthritis treatment, this study presents the development and validation of an ultra-performance liquid chromatography with quadrupole detector mass spectrometry (UPLC-QDa) method for the simultaneous quantification of tazarotene and tazarotenic acid, its active metabolite, in porcine skin. Method development involved a design-of-experiments approach for chromatographic optimization of gradient steepness, organic solvent volume, column temperature, capillary voltage, flow rate, and cone voltage. Central composite orthogonal design was used to optimize peak area, peak width, retention time, and resolution. Validation was performed in accordance with U.S. Food and Drug Administration guidelines. The method was linear over the concentration range of 0.4–18,750 ng/mL for tazarotene and 13.3–12,500 ng/mL for tazarotenic acid, with r^2^ values of ≥0.99. Chromatographic analysis demonstrated acceptable accuracy and precision (<15%), and stability tests confirmed the analytes’ stability under various conditions. This validated method offers a reliable and accurate approach for the simultaneous analysis of tazarotene and tazarotenic acid, facilitating further research into their therapeutic applications for hand osteoarthritis.

## 1. Introduction

Tazarotene, a third-generation retinoid known for its use in treating acne vulgaris and psoriasis, has gained attention as a potential pharmaceutical agent for the treatment of hand osteoarthritis [1,2,3]. This interest stems from the recognized association between the disease and the retinoic acid pathway [1]. Presently, no disease-modifying drugs are available for hand osteoarthritis, emphasizing the significant unmet need in treatment of the disease [4]. Osteoarthritis, especially of the hands, imposes a considerable burden on quality of life due to pain, stiffness, and progressive joint damage, further highlighting the need for innovative therapeutic approaches [5].

Tazarotene’s active metabolite, tazarotenic acid, binds to retinoic acid receptors (RARs), facilitating the modulation of cellular processes including differentiation, proliferation, and inflammation [6,7]. Although tazarotene may cause skin irritation, it is generally considered to be better tolerated than first- or second-generation retinoids [8]. This is believed to be largely due to the drug’s selective affinity for RAR-β and RAR-γ [9]. However, retinoic acid-derived drugs such as tazarotene are associated with considerable toxicity when used orally, including liver toxicity, teratogenicity, and dyslipidemia [9,10]. Transdermal delivery systems offer the advantage of bypassing systemic side effects commonly associated with oral administration, making them particularly advantageous for conditions like hand osteoarthritis, where targeted treatment is desirable [11]. Hand osteoarthritis activity typically affects only a select few joints at any given time, making localized treatment beneficial for addressing symptomatic joints [5,12]. By focusing on the most-impacted joints and alleviating inflammation, structural damage to the joints could be mitigated.

To assess the drug’s suitability for transdermal treatment, the permeability of the drug needs to be established by quantifying the amount of analyte retained in the skin and the amount that permeates.

The lipophilic moieties of tazarotene, such as its thiochroman ring, confer low solubility in aqueous solvents [13,14], posing challenges for some quantification methods [15]. Several liquid chromatography methods for the quantification of tazarotene and tazarotenic acid have been described [16,17,18,19,20]. While the majority focus on analyzing the analytes in plasma samples or topical formulations [16,17,19,20], one notable study investigated mini-pig skin [18]. Predominantly, these methods were developed using either reversed-phase high-performance liquid chromatography (RP-HPLC) or liquid chromatography–tandem mass spectrometry (LC-MS/MS) [16,17,18,19,20]. However, no method to date has been described or validated for the quantification of tazarotene and tazarotenic acid using ultra-performance liquid chromatography with quadrupole detector mass spectrometry (UPLC-QDa). UPLC-QDa is an appealing choice for laboratory applications as it offers a simpler, cost-effective alternative to traditional mass spectrometry, reducing the need for highly specialized equipment while maintaining analytical precision and sensitivity [21].

In this study, we address the gap in analytical methodologies by developing, optimizing, and validating a sensitive UPLC-QDa method for the simultaneous quantification of tazarotene and tazarotenic acid, using ketoconazole as the internal standard (IS). Method development and optimization followed design-of-experiments (DoE) principles, and validation adhered to U.S. Food and Drug Administration (FDA) guidelines for bioanalytical method validation [22].

## 2. Results and Discussion

### 2.1. Method Development and Optimization by Design of Experiments (DoE)

The DoE screening revealed notable differences in retention time when using acetonitrile or methanol as organic phases. Acetonitrile exhibited a significantly lower retention time compared to methanol, indicating its potential for faster chromatographic separation. Additionally, the use of acetonitrile resulted in a significantly lower back pressure compared with methanol, at around 340 bar and 715 bar, respectively. Interestingly, varying the concentration of ammonium acetate in the aqueous phase did not yield significant effects on retention time or peak asymmetry. While no significant difference was observed between ammonium acetate concentrations, 5 mM and 7.5 mM appeared to have better peak asymmetry compared to 2.5 mM ammonium acetate. Further, the 5 mM concentration demonstrated slightly superior peak asymmetry relative to the 7.5 mM concentration.

Using the central composite orthogonal design (CCO) design, method optimization was carried out and included a total of 12 responses. In the multi-linear regression analysis (Table 1), the fraction of variance explained (R2) exceeded 87% across all measured responses, with respective predictive relevance (Q2) values generally over 93%, except for ketoconazole peak width at 81.3%.

**Figure 1:**
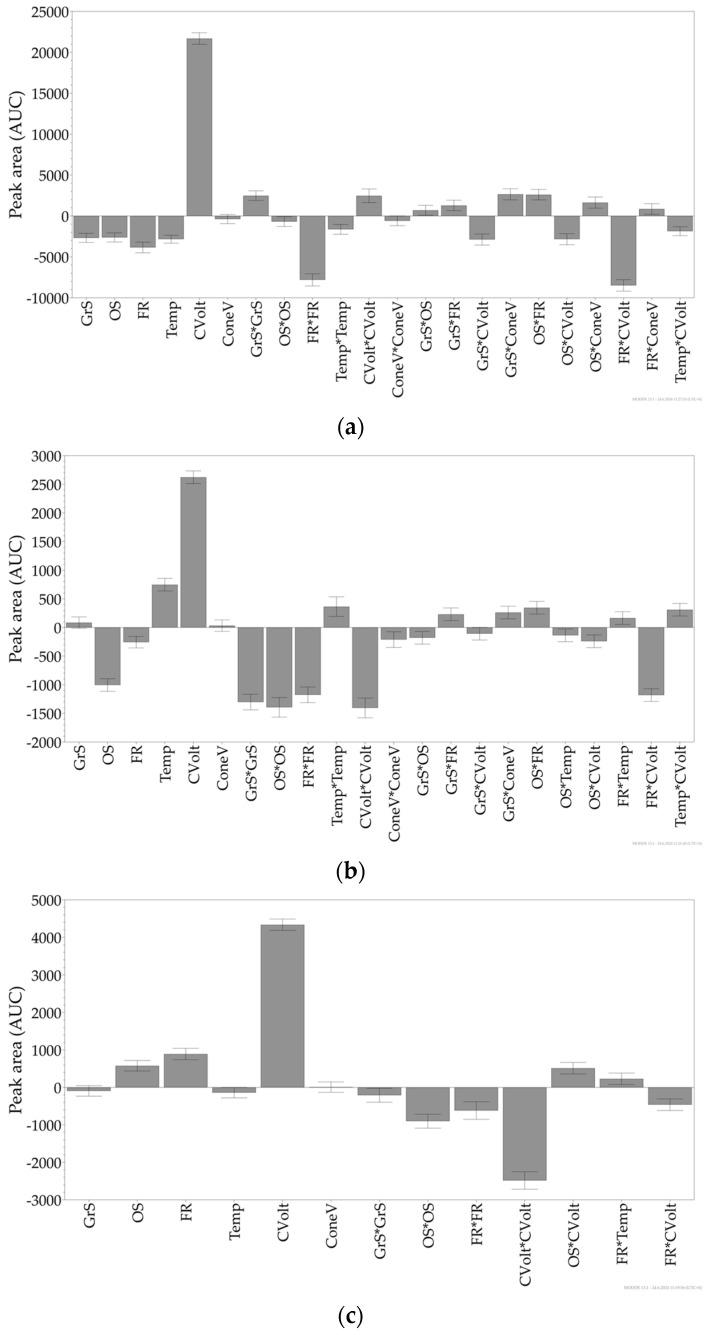
Coefficient charts from the central composite orthogonal design for the peak area of tazarotene (Area1) (**a**), tazarotenic acid (Area2) (**b**), and ketoconazole (Area3) (**c**). The x-axis represents the experimental factors as well as their interactions, while the y-axis represents the coefficient value, reflecting each factor’s effect on the response. The error bars indicate the 95% confidence interval, * represents multiplication sign. Abbreviations: GrS = gradient steepness; OS = amount of organic solvent; FR = flow rate; Temp = colum temperature; CVolt = capillary voltage; ConeV = cone voltage.

The results show that increasing the capillary voltage results in a larger peak area for all analytes. The amount of organic solvent, flow rate, gradient steepness, and column temperature all significantly negatively affected Area1, indicating that reducing these factors would result in a larger peak area of tazarotene (Figure 1a). For tazarotenic acid, on the other hand, column temperature positively affected the analyte’s peak area, while the amount of organic solvent and flow rate negatively affected it, just as with tazarotene (Figure 1b). When examining the effects that experimental factors had on tR1 and tR2, it was noted that the flow rate and the amount of organic solvent both had a significant negative effect. This indicates that reducing the flow rate and amount of organic solvent significantly increases the retention time of tazarotene and tazarotenic acid. The amount of organic solvent, gradient steepness, column temperature, and flow rate all significantly reduced Res1 and Res2. Additionally, a significant negative effect on Res1 was attributed to the capillary voltage.

**Figure 2:**
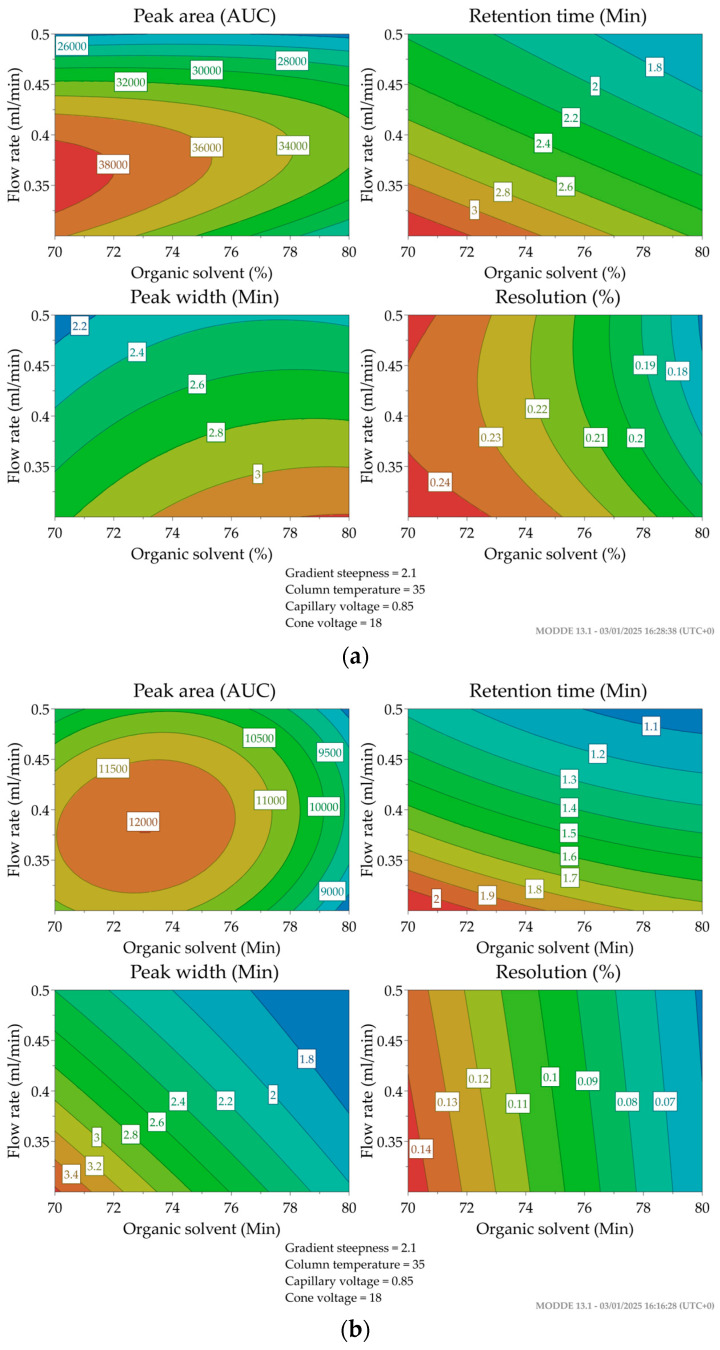
Contour plots of chromatographic response variables by flow rate and organic solvent percentage for each of the two analytes. The color scale ranges from blue to red, with red representing the highest values and dark blue the lowest. (**a**) Plots of tazarotene peak area (Area1), peak width (Width1), retention time (tR1), and resolution (Res1). (**b**) Plots of tazarotenic acid peak area (Area2), peak width (Width2), retention time (tR2), and resolution (Res2).

A number of different two-factor interactions were detected, primarily involving tazarotene and tazarotenic acid. The most frequent two-factor interaction detected was flow rate and amount of organic solvent, which was observed for Area1, tR1, Width1, Res1, Area2, tR2, Width2, Res2, and tR3. Interactions of flow rate and capillary voltage, as well as of capillary voltage and cone voltage, were the second most frequently observed interactions. Several significant quadratic effects were observed, including flow rate for the retention time and peak area of all analytes, as well as Res1, Width2, and Width3. The quadratic effect for amount of organic solvent was significant for Area1, Area2, and Area3, as well as Res1 and tR3. The contour plots in Figure 2 demonstrate the optimized conditions for tazarotene and tazarotenic acid in regard to flow rate and amount of organic solvent.

The coefficient plots demonstrate that, with the exception of cone voltage, every experimental factor showed statistical significance for at least one chosen response. The analysis, however, revealed that cone voltage did not have any significant effect on any response for any analyte. This suggests that variations in cone voltage do not influence the outcomes measured, indicating its relative insignificance in the experimental setup compared to other factors.

The optimal setting for the gradient steepness did not require any trade-off, as the factor significantly negatively affected Area1, Res2, and Res3, while having a significant positive effect on tR1 and Width1. For this reason, the lowest gradient was selected. The remaining four significant factors, however, required compromise. For the amount of organic solvent, a decrease in the experimental factor would increase Area1, Area2, and the resolution of all analytes, while at the same time decreasing Width1 and tR3. However, it would increase tR1, tR2, and Width2. With peak area and resolution being prioritized over retention time, the choice was made to opt for the lowest organic solvent amount. To attain a shorter retention time and reduced peak width, a higher flow rate is preferable. Although increasing the flow rate can lower the resolution of analytes, the positive effects on retention time and peak width are far more valuable. Consequently, a flow rate of 0.5 mL/min was chosen as part of the optimized protocol. Lowering the column temperature would increase tR1 and tR2 and decrease Area2, while at the same time increasing Area1 and Res1, Res2, and Res3. Thus, an intermediate column temperature (35 °C) was chosen for the optimized protocol. Increasing the capillary voltage would lead to a higher Area1, Area2, and Area3 while reducing Res1 and Res3. Since capillary voltage had the strongest effect on the peak area of the analytes, the highest setting (1.2 kV) was selected for the optimal method.

The optimal proportions in terms of retention time, peak shape, and resolution were found with the following settings: 70% organic solvent, gradient steepness of 1.6 min, flow rate of 0.5 mL/min, column temperature of 35 °C, and capillary voltage of 1.2 kV. As the cone voltage did not have any significant effect on any response, a default setting of 15 V was selected.

**Figure 3:**
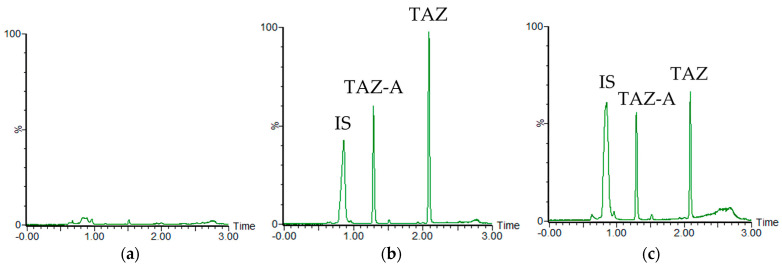
UPLC-QDa chromatograms of porcine skin samples. (**a**) blank; (**b**) skin sample with tazarotene (TAZ) and tazarotenic acid (TAZ-A) spiked with the IS; (**c**) blank spiked with tazarotene, tazarotenic acid, and the IS.

The DoE optimization achieved a resolution of >1.5 between tazarotene and tazarotenic acid. The chromatograms of the analytes obtained with the optimized method are displayed in Figure 3.

### 2.2. Method Validation Results

#### 2.2.1. Specificity

As shown in Figure 3, there were no interference peaks from impurities, degradation products or endogenous components in the retention times of the analytes. Good chromatographic separation was achieved for the analytes (tazarotene and tazarotenic acid) and the IS under the optimized UPLC-QDa conditions. The retention times of the IS, tazarotenic acid, and tazarotene were 0.84, 1.29, and 2.09 min, respectively.

#### 2.2.2. Sensitivity, Linearity, and Lowest Limit of Quantification (LLOQ)

The lowest limit of quantification (LLOQ) represents the lowest analyte concentration that can be quantified with acceptable accuracy and precision, ensuring reliable and reproducible measurements at this level. The limit of detection (LOD) and LLOQ for tazarotene were 0.10 ng/mL and 0.40 ng/mL, respectively, while the LOD and LLOQ for tazarotenic acid were 3.33 ng/mL and 13.32 ng/mL, respectively.

The results indicated a linear relationship in the range of 0.4 to 12,500 ng/mL for tazarotene and 13.3–12,500 ng/g for tazarotenic acid, demonstrating the method’s applicability for a wide range of concentrations. All linearity assessments showed a coefficient of correlation (r^2^) greater than 0.99, confirming that the method meets established criteria for analytical robustness and reliability in quantitative analysis.

#### 2.2.3. Accuracy and Precision

As shown in Table 2, the determined precisions and accuracies were within acceptable ranges (±15%, or ±20% for LLOQ). These findings confirm that the developed method is both reproducible and accurate for the simultaneous quantification of tazarotene and tazarotenic acid in porcine skin. This demonstrates the method’s reliability and suitability for use in further analytical applications involving these compounds.

#### 2.2.4. Matrix Effect

The matrix effect of the developed method ranged from 100.8% to 118.0% for both analytes (Table 3) and was consistent across high and low concentrations, with coefficient of variation (%CV) values ranging from 2.4% to 9.3%. This indicates that the sample analysis is reliable and reproducible across the entire concentration range and that the method can accurately quantify the analytes even in the presence of the skin matrix.

#### 2.2.5. Dilution Integrity

The %bias for the dilutions falls within the acceptance range of ±15%, varying from 0.96% to 10.55% for all analytes. The %CV for the dilutions, ranging from 1.3% to 7.7%, also falls below the maximum accepted level of 15%. This confirms that the accuracy and precision of measurement remain unaffected even when samples are diluted beyond the highest calibration standard, thus demonstrating that dilution does not adversely affect measurement.

#### 2.2.6. Stability

Short-term temperature stability was determined by analyzing quality control (QC) samples (at two concentrations) kept at room temperature (RT) for 8 h, and autosampler stability was assessed by analyzing samples kept under autosampler conditions (10 °C) for 24 h. Finally, the long-term stability was assessed after storage at −20 °C for 7 days. The results of the stability study are presented in Table 4, and show that the analytes remained stable under all analyzed storage conditions. The presented method is thus suitable for both short- and long-term storage.

## 3. Materials and Methods

### 3.1. Materials and Reagents

Tazarotene and tazarotenic acid were purchased from Tokyo Chemical Industry (TCI, Tokyo, Japan) and Sigma Aldrich (Taufkirchen, Germany), respectively. Formic acid (HPLC-grade) was purchased from Fluorochem (Hadfield, UK), and acetonitrile (HPLC-grade), methanol (HPLC grade), and ammonium acetate were purchased from Honeywell Riedel-de Haen (Seelze, Germany). Ketoconazole, used as the IS, was purchased from Merck (Darmstadt, Germany). All chemicals used in this study had a purity of ≥98%.

### 3.2. Study Design and Preparation of Skin Samples

In vitro permeability studies were performed using Franz diffusion cells and porcine skin. Among various animal models, porcine skin is widely valued for its structural similarity to human skin, making it a more ethically acceptable choice for use in research [23,24,25]. Fresh porcine ears, obtained as by-products from a local abattoir (Stjörnugrís, Reykjavík, Iceland), were sourced from 6-month-old pigs. Prior to use, the ears were thoroughly rinsed with deionized water and dried, and the skin was removed from the underlying cartilage. The skin was then cut into disks of radius 1.5 cm and trimmed to a thickness of approximately 1 mm using a surgical blade [26].

The porcine skin was used for the release study of tazarotene and tazarotenic acid. The drug was applied to the skin in a donor chamber. After 4 h, skin samples were collected, cleaned using purified water to remove residual drug from the exterior, and dried carefully. The skin samples were then placed in individual Eppendorf tubes and 1 mL of methanol was added for extraction. The Eppendorf tubes were then placed on a shaker at moderate speed for approximately 16 h. After this period, the extracts were filtered through a 0.45 µm syringe filter, and aliquots of the extractions were quantified using UPLC-QDa analysis.

### 3.3. Preparation of Stock Solutions and Blank Skin Matrix

Stock solutions of tazarotene (1 mg/mL) were prepared by weighing 5 mg of tazarotene and dissolving it in acetonitrile in 5 mL volumetric flasks. Similarly, stock solutions of tazarotenic acid (0.5 mg/mL) were prepared by weighing 2.5 mg of tazarotenic acid and dissolving it in acetonitrile in separate 5 mL volumetric flasks. Both solutions were stored in a freezer (−20 °C) between uses. An IS master stock solution (1 mg/mL) was prepared by weighing 10 mg of ketoconazole and dissolving it in acetonitrile in a 10 mL volumetric flask.

The blank skin matrix was prepared by extracting blank porcine skin disks in methanol for approximately 16 h on a shaker. Following the extraction period, the skin extract was filtered using a 0.45 µm syringe filter and stored in a refrigerator at 4 °C until use.

### 3.4. Preparation of Quality Control (QC) Samples and Calibration Standards

Working standard solutions were obtained by diluting stock solutions with acetonitrile to achieve concentrations of 0.1 mg/mL for tazarotene and tazarotenic acid and 0.0275 mg/mL for the IS. Once prepared, the solutions were stored at −20 °C away from light until use. The calibration standards were prepared by spiking blank skin matrix with the appropriate amounts of the working standard solutions. The prepared calibration standards were in the range of 0.40–3295 ng/mL for tazarotene and 3.33–3409 ng/mL for tazarotenic acid. Following the same process, the QC samples were prepared at four concentration levels: LLOQ, low QC (LQC), medium QC (MQC), and high QC (HQC). The concentrations of the QC samples were 0.40, 6.44, 412, and 3295 ng/mL for tazarotene, and 13.3, 53.3, 852.2, and 3409 ng/mL for tazarotenic acid.

### 3.5. Instruments and UPLC-QDa Analysis Conditions

UPLC-QDa analyses were performed on an ACQUITY UPLC H-Class System and ACQUITY QDa Mass Detector along with a PDA detector (Waters Corporation, Milford, MA, USA). For the chromatographic separation, a Hypersil Gold C18 Selectivity column (1.9 µm, 150 × 2.1 mm) was used (Thermo Fisher Scientific, Waltham, MA, USA) and the injection volume was set to 5 µL. Mobile phases A and B were composed of acetonitrile and 0.1% formic acid with 5 mM ammonium acetate (pH 3.0), respectively. Electrospray ionization (ESI) was performed in positive ionization mode using selected ion recording (SIR). Data analysis and processing were carried out using Masslynx 4.2 and Targetlynx applications (Waters Corporation, Milford, MA, USA). The amount of organic solvent, gradient steepness, flow rate, column temperature, capillary voltage, and cone voltage were optimized using DoE principles.

### 3.6. Design of Experiments

DoE screening was performed to determine the ideal mobile phases for chromatographic separation. The experimental screening by full factorial design was created in Modde 13.1 (Sartorius Data Analytics, Umeå, Sweden; latest update: 28 March 2024). The experimental factors under investigation included the choice of organic solvent (methanol vs. acetonitrile) and various concentrations of ammonium acetate in the aqueous solvent (2.5 mM, 5.0 mM, and 7.5 mM). The objective of the screening was to determine the ideal mobile-phase composition to minimize retention time and achieve symmetrical peaks with minimal fronting or tailing. Accordingly, peak asymmetry and retention time were evaluated as response parameters to assess the efficacy of each mobile-phase composition.

Once the best-performing mobile-phase combination was selected, the following factors were optimized with CCO design: capillary voltage (CVolt), column temperature (Temp), flow rate (FR), cone voltage (ConeV), gradient steepness (GrS), and amount of organic solvent (%OS). The experimental factors and their respective levels are shown in Table 5. The remaining UPLC-QDa parameter settings were set as described in Section 3.5. Analyzed responses included peak area (AUC), retention time (t_R_), peak width at half height (W_0.5_), and resolution (R_s_). Resolution was assessed for separation between ketoconazole (peak 1) and tazarotenic acid (peak 2), as well as tazarotenic acid (peak 2) and tazarotene (peak 3), and was calculated using Equation (1).

(1) $$R_{s}=1.18\times \frac{t_{R}2-t_{R}1}{W_{0.5}1+W_{0.5}2}$$

Multi-linear regression was used to analyze the relationship between the experimental factors and the UPLC-QDa responses.

### 3.7. Method Validation

Following the U.S. FDA guidelines for bioanalytical method validation [22], the UPLC-QDa method was validated for the following parameters: sensitivity; selectivity and specificity; matrix effect; accuracy and precision; dilution integrity; and stability.

#### 3.7.1. Selectivity and Specificity

Selectivity and specificity were determined by comparing blank skin samples spiked with tazarotene, tazarotenic acid, and the IS to non-spiked blank skin samples, as well as skin samples collected after 4 h of exposure to tazarotene and tazarotenic acid from six sources each (*n* = 6). This was performed to confirm the absence of interfering compounds with similar retention times to the analytes and IS.

#### 3.7.2. Sensitivity, Linearity, and LLOQ

The LOD was defined as the smallest detectable analyte concentration producing a signal-to-noise (S/N) ratio of at least 3:1, thus ensuring reliable differentiation from background noise. Sensitivity was evaluated using the LLOQ, defined as the smallest analyte amount providing a 5:1 S/N ratio which could be quantified with acceptable accuracy and precision (within ±20% of nominal concentration). This was performed using six replicates (*n* = 6) in three independent runs. Linearity was determined by weighted (1/x^2^) least-squares linear regression, plotting the peak area ratios of analytes to IS against analyte concentrations. The calibration curve’s accuracy was measured as the coefficient of determination (r^2^), with an acceptance criterion of r^2^ ≥ 0.99.

#### 3.7.3. Accuracy and Precision

To assess intraday accuracy and precision, repeated analyses (*n* = 3) were performed on QC samples at various concentrations (LLOQ, LQC, MQC and HQC) within the same day using six replicates per QC level. Interday accuracy and precision were evaluated by analyzing QC samples at the same concentration levels (*n* = 6) across three consecutive days. Accuracy was assessed based on the %bias, while precision was determined using the %CV. The acceptance criteria specified that %bias must stay within ±15% of nominal concentration, with an extended range of ±20% for LLOQ, and that %CV should not exceed these thresholds.

#### 3.7.4. Matrix Effect

The matrix effect was assessed at two QC concentrations (LQC and MQC) using six replicates (*n* = 6). Evaluation of matrix effects involves comparing the analyte in the presence of the biological matrix (analyte/IS peak area ratio) to its response in neat solution (acetonitrile). This establishes whether components in the biological matrix interfere with the accurate measurement of the analyte. The matrix effect was considered acceptable if the %bias and %CV of the peak area ratios between the matrix and neat solution were within ±15% and ≤15%, respectively.

#### 3.7.5. Dilution Integrity

For dilution integrity, blank matrix samples (*n* = 5) were spiked with analyte concentrations exceeding the highest calibration standard and diluted sevenfold with blank matrix. The measured concentration of the diluted sample was compared to the expected concentration given the dilution factor. The dilution integrity was considered acceptable if the recovery was within an acceptable range, defined as a %bias inside ±15% and a %CV under 15% of nominal concentration.

#### 3.7.6. Stability

The stabilities of tazarotene and tazarotenic acid in porcine skin were evaluated using QC samples at two concentration levels (LQC and HQC) with three replicates (*n* = 3) after storage in the following conditions: −20 °C for 7 days, post-preparation at 10 °C (autosampler conditions) for 24 h, and RT for 8 h. For samples to be considered stable, %bias had to be within ±15%.

## 4. Conclusions

In this study, a novel UPLC-QDa method was successfully developed for the simultaneous quantification of tazarotene and its active metabolite, tazarotenic acid, in porcine skin. The UPLC-QDa system provides advantages, particularly in simplifying the analytical workflow and reducing instrument cost, without compromising analytical accuracy. This is the first reported method of its kind for these analytes, and method validation demonstrated that it is highly selective, precise, accurate, linear, and robust. The validation results also confirmed the method’s specificity, with no interference from impurities or degradation products, ensuring reliable quantification.

The primary limitation of this study is that it focuses solely on porcine skin as the biological matrix, which, while a well-established model for human skin, may not allow direct translation of performance to other matrices or broader pharmacokinetic studies. Despite this, the demonstrated performance of this method provides a strong foundation for future work, including its adaptation to other biological samples. The method shows great potential for future research in the optimization of transdermal drug delivery systems and can be applied to advance therapeutic development for hand osteoarthritis. Furthermore, its sensitivity and reproducibility make it a valuable tool for broader pharmacokinetic and dermatopharmacologic studies, potentially extending its application to other dermal disorders and treatment modalities.

## Figures and Tables

**Table 1:** Abbreviations, fraction of variance explained (R2), and respective predictive relevance (Q2) values for all responses included in the design-of-experiments (DoE) optimization.

Response	Abbr.	R2	Q2
Tazarotene peak area	Area1	0.998	0.972
Tazarotene retention time	tR1	0.999	0.999
Tazarotene peak width	Width1	0.979	0.952
Resolution tazarotenic acid and tazarotene	Res1	0.988	0.965
Tazarotenic acid peak area	Area2	0.996	0.985
Tazarotenic acid retention time	tR2	0.999	0.999
Tazarotenic acid peak width	Width2	0.993	0.969
Resolution ketoconazole and tazarotenic acid	Res2	0.998	0.989
Ketoconazole peak area	Area3	0.99	0.986
Ketoconazole retention time	tR3	0.999	0.999
Ketoconazole peak width	Width3	0.875	0.813
Resolution ketoconazole and tazarotene	Res3	0.978	0.939

**Table 2:** Validation results for intra- and interday accuracy and precision.

Analyte		Intraday		Interday	
		Accuracy (%Bias)	Precision (%CV)	Accuracy (%Bias)	Precision (%CV)
Tazarotene	LLOQ	13.99	19.78	18.98	15.59
	LQC	4.73	5.80	7.97	10.08
	MQC	8.86	6.98	9.41	7.67
	HQC	5.94	7.66	4.64	6.51
Tazarotenic acid	LLOQ	10.15	5.37	10.78	11.42
	LQC	8.42	6.05	7.79	3.10
	MQC	1.96	2.81	2.24	2.69
	HQC	2.77	1.30	4.38	4.30

**Table 3:** Validation results for matrix effect.

Analyte	Concentration (ng/mL)	Mean%
		Matrix Effect
Tazarotene	LQC	103.9 ± 2.4
	MQC	111.2 ± 5.0
Tazarotenic acid	LQC	118.0 ± 9.3
	MQC	101.8 ± 4.4

**Table 4:** Assessment of tazarotene and tazarotenic acid stability under different storage conditions.

Analyte	Concentration (ng/mL)	%Bias		
		RT (8 h)	Autosampler (24 h)	−20 °C (7 d)
Tazarotene	LQC	3.37	6.79	13.74
	HQC	3.38	5.02	1.63
Tazarotenic acid	LQC	10.38	10.34	0.25
	HQC	2.49	6.56	14.52

**Table 5:** Experimental factors and their respective levels for the optimization process using the design-of-experiments (DoE) principle, performed with Modde 13.1 software.

Experimental Factors	Abbreviations	Experimental Levels		
		(−)	(0)	(+)
Gradient steepness (min)	GrS	1.6	2.1	2.6
Amount of organic solvent (%)	%OS	70	75	80
Flow rate (mL/min)	FR	0.3	0.4	0.5
Column temperature (°C)	Temp	30	35	40
Capillary voltage (kV)	CVolt	0.5	0.8	1.2
Cone voltage (V)	ConeV	15	18	21

## Data Availability

The data presented in this study are available upon request from the corresponding author. The data are not publicly available at this time as they will be used in other ongoing studies.
